# Crystal structure of 2-(*p*-tol­yl)-6-(tri­fluoro­meth­yl)benzo[*b*]thio­phene-3-carbo­nitrile

**DOI:** 10.1107/S2056989015008671

**Published:** 2015-05-09

**Authors:** N. C. Sandhya, S. Naveen, N. K. Lokanath, S. Ananda

**Affiliations:** a Department of Studies in Chemistry, University of Mysore, Manasagangotri, Mysore 570 006, India; b Institution of Excellence, University of Mysore, Manasagangotri, Mysore 570 006, India; c Department of Studies in Physics, University of Mysore, Manasagangotri, Mysore 570 006, India

**Keywords:** crystal structure, benzo[*b*]thio­phene, hydrogen bonding

## Abstract

In the title compound, C_17_H_10_F_3_NS, the dihedral angle between the fused benzo­thio­phene ring system (r.m.s. deviation = 0.042 Å) and the benzene ring is 29.78 (11)°. The crystal structure features C—H⋯F and very weak C—H⋯N hydrogen bonds, which generate (001) sheets.

## Related literature   

For background to benzo­thio­phene derivatives, see: Bettinetti *et al.* (2002 ▸); Roberts & Hartley (2004 ▸).
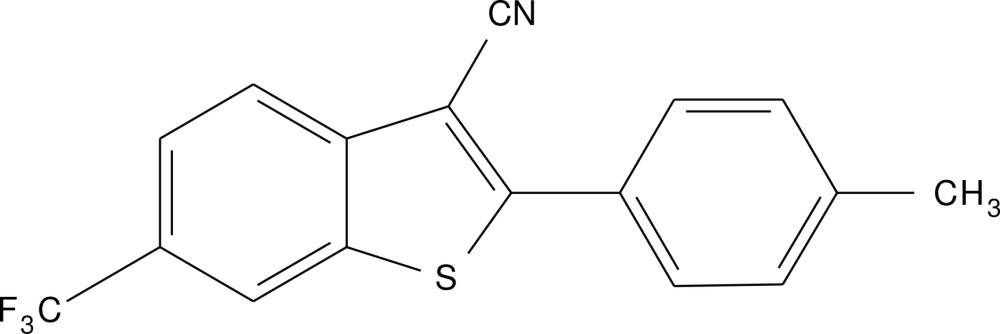


## Experimental   

### Crystal data  

C_17_H_10_F_3_NS
*M* *_r_* = 317.33
Monoclinic, 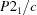

*a* = 13.7576 (5) Å
*b* = 14.5343 (6) Å
*c* = 7.1353 (3) Å
β = 92.817 (3)°
*V* = 1425.03 (10) Å^3^
*Z* = 4
Cu *K*α radiation
μ = 2.29 mm^−1^
*T* = 293 K
0.30 × 0.27 × 0.25 mm

### Data collection  

Bruker X8 Proteum diffractometer
Absorption correction: multi-scan (*SADABS*; Bruker, 2013 ▸) *T*_min_ = 0.546, *T*_max_ = 0.598
7045 measured reflections
2316 independent reflections
1860 reflections with *I* > 2σ(*I*)
*R*_int_ = 0.063

### Refinement  

*R*[*F* ^2^ > 2σ(*F* ^2^)] = 0.053
*wR*(*F* ^2^) = 0.141
*S* = 1.06
2316 reflections
200 parameters
H-atom parameters constrained
Δρ_max_ = 0.43 e Å^−3^
Δρ_min_ = −0.40 e Å^−3^

### 

Data collection: *APEX2* (Bruker, 2013 ▸); cell refinement: *SAINT* (Bruker, 2013 ▸); data reduction: *SAINT*; program(s) used to solve structure: *SHELXS97* (Sheldrick, 2008 ▸); program(s) used to refine structure: *SHELXL97* (Sheldrick, 2008 ▸); molecular graphics: *PLATON* (Spek, 2009 ▸); software used to prepare material for publication: *PLATON*.

## Supplementary Material

Crystal structure: contains datablock(s) global, I. DOI: 10.1107/S2056989015008671/hb7416sup1.cif

Structure factors: contains datablock(s) I. DOI: 10.1107/S2056989015008671/hb7416Isup2.hkl

Supporting information file. DOI: 10.1107/S2056989015008671/hb7416Isup3.cml

**Fig. 1.**
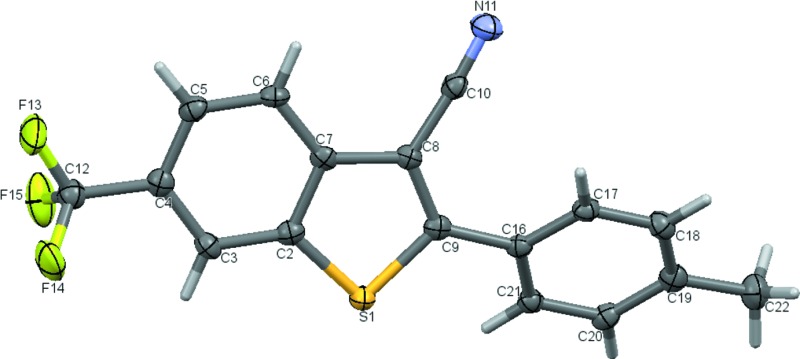
A view of the title compound with displacement ellipsoids drawn at the 50% probability level.

**Fig. 2.**
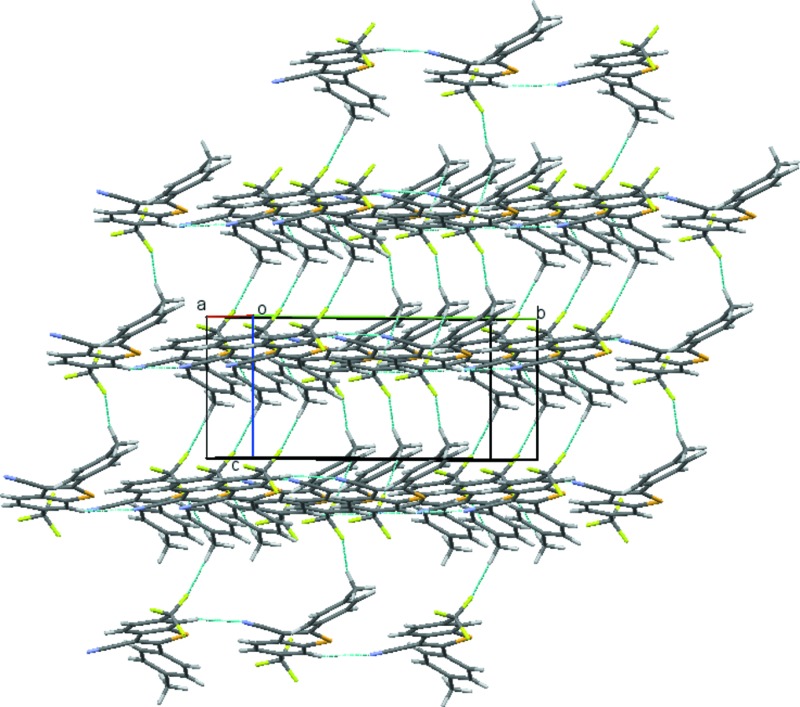
A view along the a axis of the crystal packing of the title compound. Hydrogen bonds are shown as dashed lines (see Table 2 for details).

. DOI: 10.1107/S2056989015008671/hb7416fig1.tif
A view of the title compound with displacement ellipsoids drawn at the 50% probability level.

a . DOI: 10.1107/S2056989015008671/hb7416fig2.tif
A view along the *a* axis of the crystal packing of the title compound. Hydrogen bonds are shown as dashed lines (see Table 2 for details).

CCDC reference: 1063141

Additional supporting information:  crystallographic information; 3D view; checkCIF report

## Figures and Tables

**Table 1:** Hydrogen-bond geometry (, )

*D*H*A*	*D*H	H*A*	*D* *A*	*D*H*A*
C3H3N11^i^	0.93	2.62	3.411(4)	143
C22H22*C*F15^ii^	0.96	2.45	3.375(4)	162

Symmetry codes: (i) 
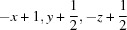
; (ii) 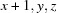.

## supplementary crystallographic information

### S1. Comment

Benzo[*b*]thiophene derivatives are important heterocyclic compounds because of their various applications in medicinal chemistry. They represent an important heterocyclic core and are shown to display a range of promising pharmacological properties such as antipsychotic, antidepressive, antithrombolytic, dopamine receptor antagonist and 5-lipoxygenase inhibitor. Number of 2-arylbenzo[*b*]thiophene derivatives have indeed, these sulfur heterocycles are essential components of clinically important drugs such as Clopidogrel (Bettinetti *et al.*, 2002), Raloxifene (Roberts & Hartley, 2004) and Zileuton.

### S2. Experimental

To a solution of 2-(2-chlorophenyl)acetonitrile (1.0 mmol), methyl benzodithioate (1.0 mmol) in DMF (2 ml), K_3_PO_4_ (2.0 mmol), pivalic acid (1.5 mmol), cuprous iodide (0.2 mmol) were added. The mixture was stirred at 80°C and progress was monitored by TLC. When the dithioesters could no longer be detected, the reaction mixture was extracted with EtOAc (3 × 10 ml). The organic layer was dried over anhydrous Na_2_SO_4_. The solvent was then removed under reduced pressure and the residue was purified by silica gel chromatography. White solid single crystals were obtained from slow evaporation of its solvent.

### S3. Refinement

All H atoms were positioned geometrically and allowed to ride on their parent atom, with C–H = 0.93–0.97 Å, and with *U*_iso_(H) = 1.2–1.5*U*_eq_(C).

### Crystal data

**Table:**

C_17_H_10_F_3_NS	*F*(000) = 648
*M**_r_* = 317.33	*D*_x_ = 1.479 Mg m^−^^3^
Monoclinic, *P*2_1_/*c*	Cu *K*α radiation, λ = 1.54178 Å
Hall symbol: -P 2ybc	Cell parameters from 2316 reflections
*a* = 13.7576 (5) Å	θ = 6.4–64.7°
*b* = 14.5343 (6) Å	µ = 2.29 mm^−^^1^
*c* = 7.1353 (3) Å	*T* = 293 K
β = 92.817 (3)°	Block, colourless
*V* = 1425.03 (10) Å^3^	0.30 × 0.27 × 0.25 mm
*Z* = 4	

### Data collection

**Table:**

Bruker X8 Proteum diffractometer	2316 independent reflections
Radiation source: Rotating Anode	1860 reflections with *I* > 2σ(*I*)
Graphite monochromator	*R*_int_ = 0.063
Detector resolution: 18.4 pixels mm^-1^	θ_max_ = 64.7°, θ_min_ = 6.4°
φ and ω scans	*h* = −15→15
Absorption correction: multi-scan (*SADABS*; Bruker, 2013)	*k* = −15→16
*T*_min_ = 0.546, *T*_max_ = 0.598	*l* = −8→6
7045 measured reflections	

### Refinement

**Table:**

Refinement on *F*^2^	Primary atom site location: structure-invariant direct methods
Least-squares matrix: full	Secondary atom site location: difference Fourier map
*R*[*F*^2^ > 2σ(*F*^2^)] = 0.053	Hydrogen site location: inferred from neighbouring sites
*wR*(*F*^2^) = 0.141	H-atom parameters constrained
*S* = 1.06	*w* = 1/[σ^2^(*F*_o_^2^) + (0.0726*P*)^2^ + 0.2459*P*] where *P* = (*F*_o_^2^ + 2*F*_c_^2^)/3
2316 reflections	(Δ/σ)_max_ < 0.001
200 parameters	Δρ_max_ = 0.43 e Å^−^^3^
0 restraints	Δρ_min_ = −0.40 e Å^−^^3^

### Special details

**Table:**

Geometry. Bond distances, angles *etc*. have been calculated using the rounded fractional coordinates. All su's are estimated from the variances of the (full) variance-covariance matrix. The cell e.s.d.'s are taken into account in the estimation of distances, angles and torsion angles
Refinement. Refinement on *F*^2^ for ALL reflections except those flagged by the user for potential systematic errors. Weighted *R*-factors *wR* and all goodnesses of fit *S* are based on *F*^2^, conventional *R*-factors *R* are based on *F*, with *F* set to zero for negative *F*^2^. The observed criterion of *F*^2^ > σ(*F*^2^) is used only for calculating -*R*-factor-obs *etc*. and is not relevant to the choice of reflections for refinement. *R*-factors based on *F*^2^ are statistically about twice as large as those based on *F*, and *R*-factors based on ALL data will be even larger.

### Fractional atomic coordinates and isotropic or equivalent isotropic displacement parameters (Å^2^)

**Table:**

	*x*	*y*	*z*	*U*_iso_*/*U*_eq_	
S1	0.51842 (5)	0.17833 (5)	0.23250 (10)	0.0214 (2)	
F13	0.10569 (13)	−0.01265 (15)	0.0632 (4)	0.0518 (8)	
F14	0.14077 (13)	0.11887 (16)	−0.0469 (3)	0.0450 (7)	
F15	0.11752 (13)	0.10360 (17)	0.2439 (3)	0.0484 (8)	
N11	0.64047 (19)	−0.14957 (19)	0.3600 (4)	0.0297 (9)	
C2	0.4251 (2)	0.0996 (2)	0.1944 (4)	0.0202 (9)	
C3	0.3272 (2)	0.1178 (2)	0.1485 (4)	0.0212 (9)	
C4	0.2634 (2)	0.0444 (2)	0.1373 (4)	0.0210 (9)	
C5	0.2957 (2)	−0.0459 (2)	0.1679 (4)	0.0217 (9)	
C6	0.3920 (2)	−0.0643 (2)	0.2103 (4)	0.0203 (8)	
C7	0.4583 (2)	0.0087 (2)	0.2249 (4)	0.0178 (8)	
C8	0.5601 (2)	0.0069 (2)	0.2816 (4)	0.0174 (8)	
C9	0.6018 (2)	0.0928 (2)	0.2949 (4)	0.0183 (8)	
C10	0.6079 (2)	−0.0785 (2)	0.3267 (4)	0.0204 (9)	
C12	0.1574 (2)	0.0636 (2)	0.0993 (4)	0.0253 (9)	
C16	0.7014 (2)	0.1180 (2)	0.3613 (4)	0.0182 (8)	
C17	0.7798 (2)	0.0588 (2)	0.3430 (4)	0.0198 (9)	
C18	0.8717 (2)	0.0826 (2)	0.4144 (4)	0.0236 (9)	
C19	0.8890 (2)	0.1655 (2)	0.5074 (4)	0.0235 (9)	
C20	0.8107 (2)	0.2255 (2)	0.5220 (4)	0.0217 (9)	
C21	0.7190 (2)	0.2025 (2)	0.4506 (4)	0.0194 (8)	
C22	0.9878 (2)	0.1887 (2)	0.5939 (5)	0.0306 (10)	
H3	0.30560	0.17760	0.12600	0.0250*	
H5	0.25120	−0.09410	0.15940	0.0260*	
H6	0.41310	−0.12450	0.22910	0.0240*	
H17	0.77040	0.00260	0.28230	0.0240*	
H18	0.92320	0.04210	0.39990	0.0280*	
H20	0.82050	0.28200	0.58110	0.0260*	
H21	0.66800	0.24390	0.46190	0.0230*	
H22A	0.99640	0.15870	0.71340	0.0460*	
H22B	0.99300	0.25410	0.61070	0.0460*	
H22C	1.03700	0.16810	0.51280	0.0460*	

### Atomic displacement parameters (Å^2^)

**Table:**

	*U*^11^	*U*^22^	*U*^33^	*U*^12^	*U*^13^	*U*^23^
S1	0.0194 (4)	0.0164 (4)	0.0281 (4)	−0.0001 (3)	−0.0009 (3)	0.0010 (3)
F13	0.0215 (9)	0.0385 (13)	0.0941 (17)	−0.0028 (9)	−0.0107 (10)	−0.0047 (12)
F14	0.0293 (10)	0.0607 (15)	0.0442 (12)	0.0123 (10)	−0.0052 (9)	0.0178 (11)
F15	0.0285 (10)	0.0782 (17)	0.0390 (11)	0.0161 (10)	0.0059 (9)	−0.0160 (11)
N11	0.0321 (15)	0.0196 (16)	0.0369 (16)	0.0022 (12)	−0.0027 (12)	0.0019 (13)
C2	0.0237 (15)	0.0198 (16)	0.0171 (14)	−0.0014 (12)	0.0024 (11)	−0.0003 (13)
C3	0.0214 (14)	0.0211 (17)	0.0210 (14)	0.0051 (12)	0.0011 (12)	−0.0005 (13)
C4	0.0223 (15)	0.0258 (17)	0.0149 (13)	−0.0007 (13)	0.0005 (11)	−0.0005 (13)
C5	0.0244 (15)	0.0211 (17)	0.0196 (14)	−0.0038 (12)	−0.0001 (12)	−0.0010 (13)
C6	0.0268 (15)	0.0145 (15)	0.0198 (14)	−0.0004 (12)	0.0026 (12)	0.0003 (12)
C7	0.0216 (14)	0.0207 (16)	0.0111 (13)	0.0011 (12)	0.0023 (11)	0.0016 (12)
C8	0.0203 (14)	0.0170 (15)	0.0149 (13)	0.0012 (12)	0.0017 (11)	−0.0007 (12)
C9	0.0220 (14)	0.0191 (16)	0.0142 (13)	0.0006 (12)	0.0041 (11)	−0.0006 (12)
C10	0.0199 (14)	0.0220 (18)	0.0191 (15)	−0.0039 (13)	−0.0011 (12)	−0.0006 (13)
C12	0.0240 (15)	0.0254 (18)	0.0265 (16)	0.0002 (13)	0.0002 (13)	−0.0017 (14)
C16	0.0206 (14)	0.0174 (16)	0.0166 (13)	−0.0004 (12)	0.0005 (11)	0.0015 (12)
C17	0.0219 (15)	0.0169 (16)	0.0209 (14)	−0.0001 (12)	0.0034 (12)	−0.0013 (13)
C18	0.0229 (15)	0.0240 (17)	0.0241 (15)	0.0042 (13)	0.0026 (12)	−0.0007 (13)
C19	0.0237 (15)	0.0293 (18)	0.0175 (14)	−0.0019 (13)	0.0003 (12)	0.0033 (13)
C20	0.0240 (15)	0.0223 (17)	0.0187 (14)	−0.0044 (13)	0.0011 (12)	−0.0009 (13)
C21	0.0204 (14)	0.0186 (16)	0.0195 (14)	0.0003 (12)	0.0032 (11)	0.0022 (13)
C22	0.0251 (16)	0.037 (2)	0.0295 (16)	0.0014 (14)	−0.0015 (13)	−0.0015 (16)

### Geometric parameters (Å, º)

**Table:**

S1—C2	1.731 (3)	C16—C17	1.391 (4)
S1—C9	1.735 (3)	C16—C21	1.399 (4)
F13—C12	1.335 (4)	C17—C18	1.384 (4)
F14—C12	1.328 (4)	C18—C19	1.390 (4)
F15—C12	1.326 (4)	C19—C20	1.394 (4)
N11—C10	1.146 (4)	C19—C22	1.503 (4)
C2—C3	1.396 (4)	C20—C21	1.378 (4)
C2—C7	1.411 (4)	C3—H3	0.9300
C3—C4	1.381 (4)	C5—H5	0.9300
C4—C5	1.399 (4)	C6—H6	0.9300
C4—C12	1.496 (4)	C17—H17	0.9300
C5—C6	1.371 (4)	C18—H18	0.9300
C6—C7	1.400 (4)	C20—H20	0.9300
C7—C8	1.439 (4)	C21—H21	0.9300
C8—C9	1.375 (4)	C22—H22A	0.9600
C8—C10	1.434 (4)	C22—H22B	0.9600
C9—C16	1.474 (4)	C22—H22C	0.9600
			
C2—S1—C9	92.44 (14)	C17—C16—C21	117.9 (3)
S1—C2—C3	127.7 (2)	C16—C17—C18	120.6 (3)
S1—C2—C7	111.3 (2)	C17—C18—C19	121.6 (3)
C3—C2—C7	121.0 (3)	C18—C19—C20	117.6 (3)
C2—C3—C4	118.1 (3)	C18—C19—C22	121.4 (3)
C3—C4—C5	121.3 (3)	C20—C19—C22	121.0 (3)
C3—C4—C12	118.5 (3)	C19—C20—C21	121.2 (3)
C5—C4—C12	120.2 (3)	C16—C21—C20	121.1 (3)
C4—C5—C6	120.9 (3)	C2—C3—H3	121.00
C5—C6—C7	119.2 (3)	C4—C3—H3	121.00
C2—C7—C6	119.6 (3)	C4—C5—H5	120.00
C2—C7—C8	111.3 (2)	C6—C5—H5	120.00
C6—C7—C8	129.0 (3)	C5—C6—H6	120.00
C7—C8—C9	113.6 (3)	C7—C6—H6	120.00
C7—C8—C10	120.6 (3)	C16—C17—H17	120.00
C9—C8—C10	125.8 (3)	C18—C17—H17	120.00
S1—C9—C8	111.4 (2)	C17—C18—H18	119.00
S1—C9—C16	119.8 (2)	C19—C18—H18	119.00
C8—C9—C16	128.7 (3)	C19—C20—H20	119.00
N11—C10—C8	175.6 (3)	C21—C20—H20	119.00
F13—C12—F14	106.3 (2)	C16—C21—H21	120.00
F13—C12—F15	106.1 (2)	C20—C21—H21	119.00
F13—C12—C4	112.7 (2)	C19—C22—H22A	109.00
F14—C12—F15	106.5 (2)	C19—C22—H22B	109.00
F14—C12—C4	112.6 (2)	C19—C22—H22C	109.00
F15—C12—C4	112.2 (2)	H22A—C22—H22B	109.00
C9—C16—C17	121.9 (3)	H22A—C22—H22C	110.00
C9—C16—C21	120.1 (3)	H22B—C22—H22C	109.00
			
C9—S1—C2—C3	175.6 (3)	C2—C7—C8—C9	0.5 (4)
C9—S1—C2—C7	−1.4 (2)	C2—C7—C8—C10	177.7 (3)
C2—S1—C9—C8	1.7 (2)	C6—C7—C8—C9	−175.0 (3)
C2—S1—C9—C16	−175.3 (2)	C6—C7—C8—C10	2.2 (5)
S1—C2—C3—C4	−175.5 (2)	C7—C8—C9—S1	−1.6 (3)
C7—C2—C3—C4	1.2 (4)	C7—C8—C9—C16	175.2 (3)
S1—C2—C7—C6	176.7 (2)	C10—C8—C9—S1	−178.6 (2)
S1—C2—C7—C8	0.8 (3)	C10—C8—C9—C16	−1.8 (5)
C3—C2—C7—C6	−0.5 (4)	S1—C9—C16—C17	−154.1 (2)
C3—C2—C7—C8	−176.4 (3)	S1—C9—C16—C21	27.8 (4)
C2—C3—C4—C5	−1.0 (4)	C8—C9—C16—C17	29.4 (5)
C2—C3—C4—C12	176.5 (3)	C8—C9—C16—C21	−148.7 (3)
C3—C4—C5—C6	0.1 (4)	C9—C16—C17—C18	−177.0 (3)
C12—C4—C5—C6	−177.4 (3)	C21—C16—C17—C18	1.2 (4)
C3—C4—C12—F13	170.3 (3)	C9—C16—C21—C20	176.8 (3)
C3—C4—C12—F14	50.1 (4)	C17—C16—C21—C20	−1.5 (4)
C3—C4—C12—F15	−70.0 (3)	C16—C17—C18—C19	0.4 (4)
C5—C4—C12—F13	−12.2 (4)	C17—C18—C19—C20	−1.8 (4)
C5—C4—C12—F14	−132.4 (3)	C17—C18—C19—C22	176.6 (3)
C5—C4—C12—F15	107.5 (3)	C18—C19—C20—C21	1.5 (4)
C4—C5—C6—C7	0.7 (4)	C22—C19—C20—C21	−176.9 (3)
C5—C6—C7—C2	−0.5 (4)	C19—C20—C21—C16	0.1 (4)
C5—C6—C7—C8	174.6 (3)		

### Hydrogen-bond geometry (Å, º)

**Table:**

*D*—H···*A*	*D*—H	H···*A*	*D*···*A*	*D*—H···*A*
C3—H3···N11^i^	0.93	2.62	3.411 (4)	143
C22—H22*C*···F15^ii^	0.96	2.45	3.375 (4)	162

Symmetry codes: (i) −*x*+1, *y*+1/2, −*z*+1/2; (ii) *x*+1, *y*, *z*.
