# Supplementary figures and images for: Crystal structure of 2-(p-tol­yl)-6-(tri­fluoro­meth­yl)benzo[b]thio­phene-3-carbo­nitrile
Source: Acta Crystallogr E Crystallogr Commun. 2015 May 9;71(Pt 6):o382. doi: 10.1107/S2056989015008671 (PMC4459355; doi:10.1107/S2056989015008671)

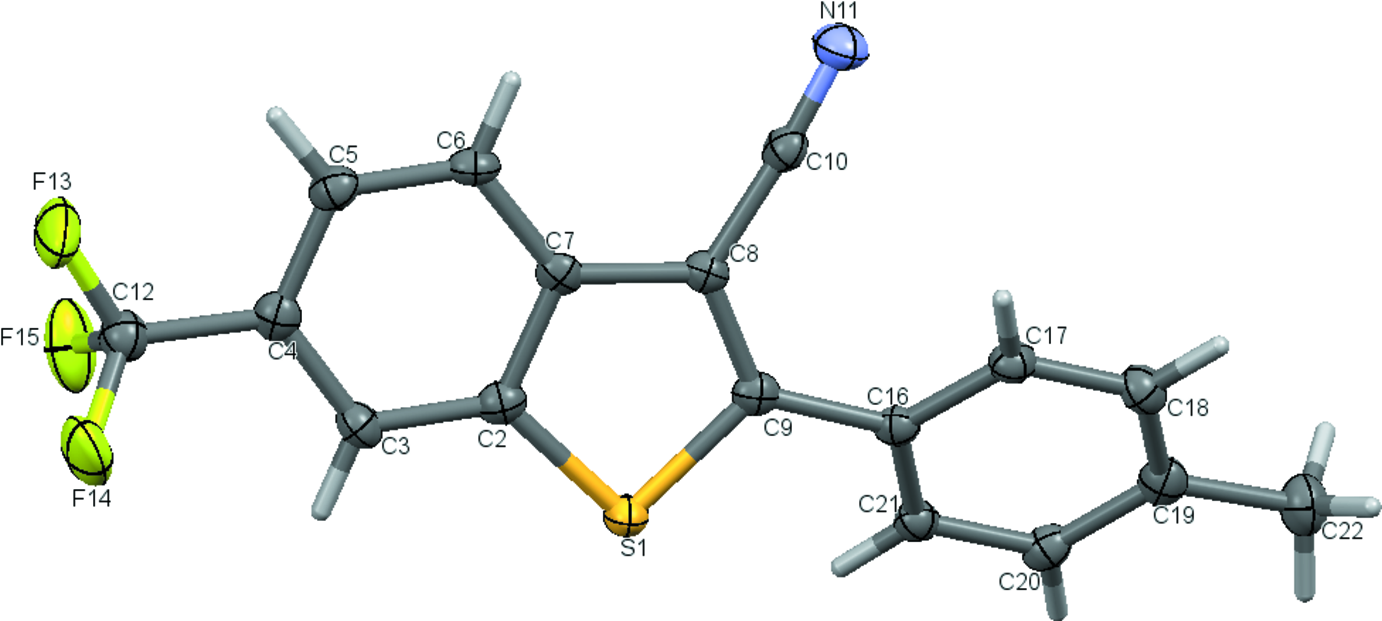

Supplement: Supplementary file 4 [file e-71-0o382-fig1.tif]

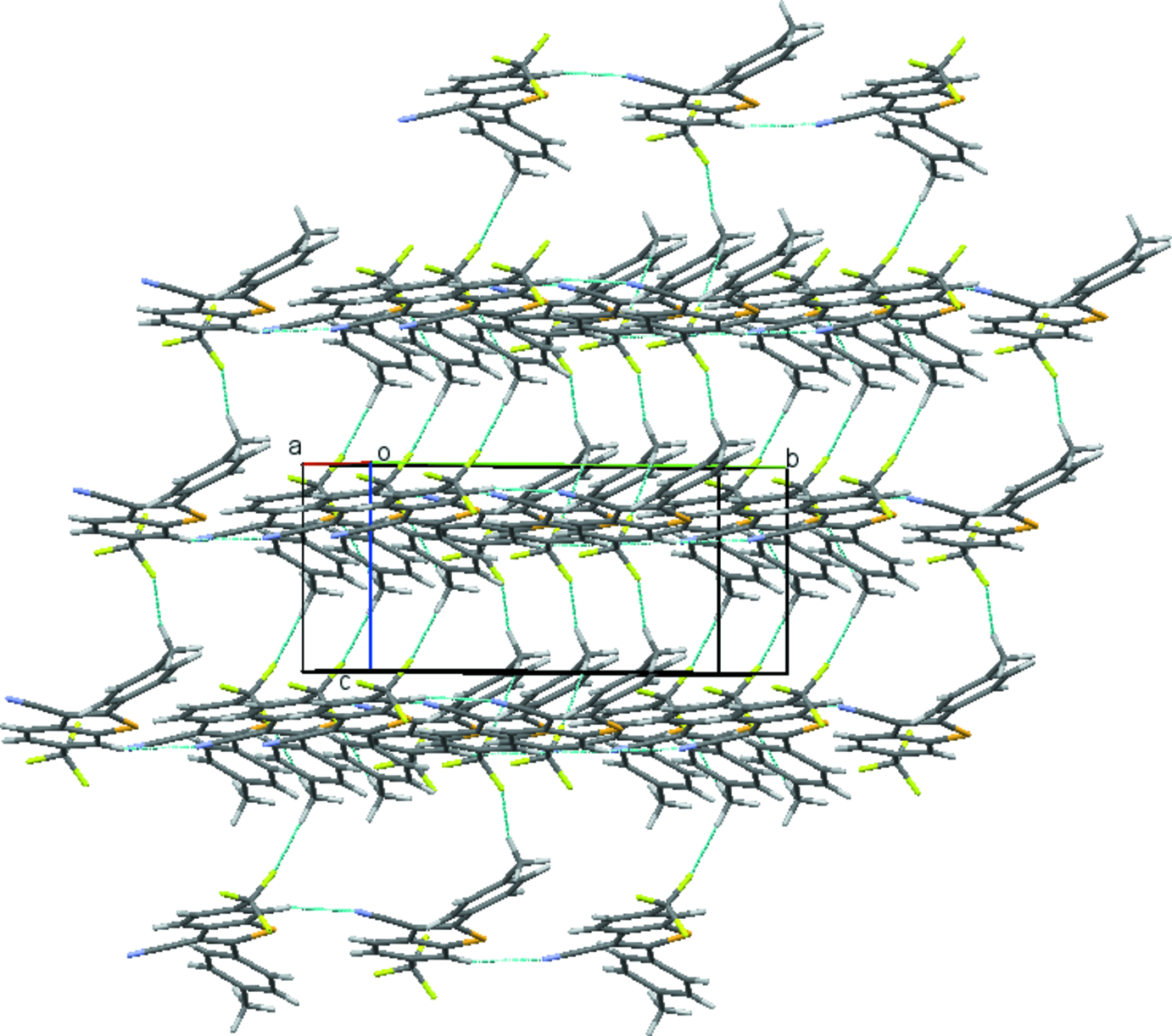

Supplement: Supplementary file 5 [file e-71-0o382-fig2.tif]
